# Donor-Site Morbidity after Fibula Transplantation in Head and Neck Tumor Patients: A Split-Leg Retrospective Study with Focus on Leg Stability and Quality of Life

**DOI:** 10.3390/cancers12082217

**Published:** 2020-08-08

**Authors:** Sameh Attia, Jonas Diefenbach, Daniel Schmermund, Sebastian Böttger, Jörn Pons-Kühnemann, Christine Scheibelhut, Christian Heiss, Hans-Peter Howaldt

**Affiliations:** 1 Department of Cranio Maxillofacial Surgery, Justus-Liebig University Giessen, Klinik Str. 33, 35392 Giessen, Germany; jdiefenbach@gmail.com (J.D.); Daniel.Schmermund@uniklinikum-giessen.de (D.S.); Sebastian.Boettger@uniklinikum-giessen.de (S.B.); hp.howaldt@uniklinikum-giessen.de (H.-P.H.); 2 Institute for Medical Informatics, Medical Statistics, Faculty of Medicine, Justus-Liebig University Giessen, Rudolf-Buchheim Str. 6, 35392 Giessen, Germany; Joern.Pons@informatik.med.uni-giessen.de (J.P.-K.); christine.scheibelhut@informatik.med.uni-giessen.de (C.S.); 3 Department of Trauma, Hand and Reconstructive Surgery, Justus-Liebig University Giessen, Rudolf-Buchheim- Str. 7, 35392 Giessen, Germany; christian.heiss@chiru.med.uni-giessen.de

**Keywords:** fibula flap, donor-site morbidity, SEBT, FADI, maxillofacial surgery, head and neck cancer, microvascular flaps

## Abstract

The free fibula flap has been one of the most important microvascular grafts for orofacial reconstruction for more than 30 years. The complication rates at the donor-site reported in literature are considered to be low, but the published data vary greatly in some cases. In particular, restrictions in the stability and balance of the involved leg and their effects on the quality of life have been described very inconsistently to date. Therefore, this study mainly focuses on the stability and balance of the affected leg in a split-leg design. Between December 2014 and January 2018, out of 119 subjects who underwent mainly jaw ablative tumor surgery and reconstruction using a fibula flap, 68 subjects were examined for donor site morbidity. Besides reporting general types of complications, two specific test procedures were used. The Star Excursion Balance Test (SEBT) as a practical test for ankle function and the Foot and Ankle Disability Index (FADI) as a questionnaire in order to assess quality of life, depending on the lower leg function. SEBT revealed an average of 55.3 cm with the operated leg as the supporting leg, which corresponds to 95.5% of 57.9 cm achieved with the healthy leg as the supporting leg. An average FADI score of 89.4% was recorded. SEBT and FADI seem to be suitable methods of examination for subjects post fibular transplantation and pointed out minimal limitations of the involved legs in comparison to the unaffected legs. These limitations were clinically not relevant and they had minor influence on the subjects’ quality of life and their daily activities.

## 1. Introduction

Since its first description over 40 years ago, the fibular graft has become one of the most important grafts in oral and maxillofacial surgery [1]. It is used for a wide range of indications, but, by far, the largest and most demanding part of fibula transplants in the orofacial area still relates to reconstructive surgery after ablative tumor resection [2]. A fibula flap was proven to be a reliable method for bone reconstruction of the jaws and it can be regarded as the workhorse for functional and aesthetic rehabilitation in oncologic patients [3,4] suffering from head and neck cancer. Worldwide, more than 300,000 patients a year are diagnosed with head and neck cancer [5]. The most common head and neck tumor is the squamous cell carcinoma, accounting for approx. 90% of cases, followed by adenocarcinoma, accounting for approx. 5% of cases [6]. The tumorigenesis has several risk factors, such as alcohol abuse, smoking, poor oral hygiene, as well as genetic aspects in rare cases [7]. Furthermore, infections with human papilloma viruses (HPV) are increasingly recognized as a key tumorigenic factor, especially when located in the area of the throat [8]. The main goals of cancer treatment are the removal of the entire tumor and subsequent reconstruction of the resected tissues [9]. Oral cancer patients often undergo extensive surgical excision of the tumor, including mandibular and/or maxillary resection. These hard and soft tissue defects lead to a series of functional and aesthetic problems [10]. The best time for jaw reconstruction in oncologic head and neck patients is the time of resection [11]. The immediate reconstruction provides perfect access to the bone margin after the ablative surgery, leading to the prompt assessment of the hard and soft tissue defects. It also reduces the number of surgical procedures and achieves oral rehabilitation in a shorter amount of time [12]. In this setting, there is also the best opportunity to find suitable vessels for the microanastomosis required for a fibular flap or any other microvascular flap. Frozen-section analysis and flat-panel volume computed tomography of the resected tissues ensure high reliability in terms of the complete removal of the tumor [13]. The immediate reconstruction of the complex soft-tissue and bone defects caused by the tumor ablative surgery requires free vascularized tissue transplant [14].

Microvascular techniques were first introduced in the 1970s and, from this time forward, several autologous grafts for reconstructive head and neck surgery have been proposed [15]. In 1975, Taylor et al. performed the first microvascular fibular bone transfer [16]. In 1983, Chen and Yan were the first to use an osteocutaneous fibula flap for the reconstruction of bone and soft tissue defects [17]. By using osteotomies in the fibular bone, Hidalgo performed the first lower jaw reconstruction to simulate the form of the mandible [18,19]. Since 1990, the fibula flap is widely used for facial reconstruction [17,20]. To overcome the limited height of the fibula, Jones et al. introduced a double barrel technique, which postulates that two osteotomized bone segments of fibula can be folded in order to cover a greater height [21].

The fibula flap is widely used and preferred in the head and neck reconstructive surgery due to several advantages. Besides the considerable length of available bone, the fibula transfer is a feasible technique and it can be performed as a two-team approach leading to a significant reduction of operation time [18]. Furthermore, the flap possesses a sufficiently long and high-caliber vascular pedicle, making microsurgical anastomoses feasible to perform [22]. Cortical bone allows for the insertion of primary stable dental implants required for dental rehabilitation [3].

Various studies in literature report on the use of fibula grafts for orofacial reconstruction in general and the associated donor-site morbidity in particular. Although these studies describe the limitations that are associated with fibula removal as minor [23,24,25], complications can still occur with severe consequences for patients [26,27,28]. The methods often differ greatly between individual studies. On the one hand, limitations were recorded subjectively by the patients in individual questions or by means of questionnaires; on the other hand, there were various practical test methods. These range from simple sensomotoric examinations of the N. fibularis and walking tests, to complex optical and kinetic analyses of leg function [29,30,31,32,33]. However, data regarding donor site morbidity after lifting of the fibula flap in a split-leg design are rare in literature [34]. The here presented study mainly focuses on the stability and balance of the affected leg. The upper ankle, including fibula, tibia, and talus in particular seemed to be an interesting indicator. To date, the effect of fibular transplantation has not been examined in detail. We have selected a specific test procedure to objectively investigate the donor-site morbidity. To determine ankle stability, we selected the Star Excursion Balance Test (SEBT), which is a clinical balance test. The Foot and Ankle Disability Index (FADI) questionnaire was selected to additionally examine the influence of ankle function on quality of life. Both tests are highly reliable and they have been proven in orthopedics and sport medicine [35,36,37]. The hypothesis of this study is that fibula transplantation only slightly restricts leg function and that the loss of quality of life due to donor site morbidity is minor/acceptable. Additionally, this study aims to answer the following questions:

- How is the ankle function and thus the balance and stability of a leg restricted by the removal of a fibular graft?
- How much does the aforementioned restriction affect daily activities and quality of life?

Answering the study questions should help to further improve patient selection and the surgical procedure itself and reduce donor site morbidity in the future.

## 2. Results

### 2.1. Basic Data

A total of 119 subjects were included according to the previously defined inclusion criteria. Fifty-one subjects could not be examined and were considered as drop-out due to the following reasons: 30 subjects had deceased; two subjects lived abroad; three subjects were in hospital care; seven subjects found themselves unable to participate due to their limited physical condition; and, nine subjects could not be reached by phone or mail. A total of 68 subjects, 22 female (32.4%) and 46 male (67.6%), were examined and included in the study, which corresponds to a rate of 57%. The mean age of the subjects at surgery was 55.4 years (median 57 years), with the youngest patient being 17 years old and the oldest patient being 80 years old. The mean time between the fibula transplantation surgery and follow-up for study subjects (*n* = 68) was 1428 days (minimum 51, median 809, and maximum 5083 days).

#### Height, Weight and Body Mass Index (BMI)

The average height was 1.74 m (median 1.78 m) and varied between 1.5 and 1.92 m, the average body weight was 73.8 kg (median 75 kg) and varied between 43 and 118 kg. On average, a body mass index of 24.3 (median 23.6) was calculated. The values fluctuated between 16.4 and 39.1 (Table 1).

### 2.2. Surgical Information

#### 2.2.1. Indications and Localization of Defects

The most common indication for a fibula transplantation was cancer resection. As the most common tumor in the head and neck area, squamous cell carcinoma clearly predominates, with 69%. From our cohort of 68 subjects, 65 had tumors, three subjects underwent surgery due to atrophy (*n* = 2) and osteoradionecrosis (*n* = 1). Almost three-quarters of the defects were located in the lower jaw (73.5% *n* = 50), less than one quarter in the upper jaw (26.5%, *n* = 18). The distribution can be explained by the fact that the fibular graft is particularly well suited for the reconstruction of the lower jaw (Table 1).

#### 2.2.2. Donor-Site, Type of Fibula Transplantation, and Wound Closure

The transplants were harvested from the left lower leg in precisely 25% of cases (*n* = 17) and from the right lower leg in 75% of cases (*n* = 51). If preoperative diagnostics, especially femoral angiography, does not conflict with this, the right leg is usually chosen for ergonomic reasons. We distinguished between three different types of grafts:

- osseomyocutan graft (bone, muscle tissue, and skin island);
- osseomuscular graft (bone and muscle tissue); and,
- prefabricated fibula graft (bone covered with pre-transplanted skin graft and already inserted dental implants) (Table 1).

Tumor reconstruction was the main indication for a fibula graft (Table 1). The operated tumors, mainly squamous cell carcinomas, usually also infiltrate adjacent tissue, which makes resection with a sufficiently large safety margin necessary. The resulting bone and soft tissue defects can be best reconstructed with a graft containing soft tissue and skin in addition to bone. For this reason, most fibular grafts are raised as osseomyocutaneous grafts. Most of the wounds at the donor site were closed using skin grafts from the thigh region (*n* = 50, 73.5%), and the remaining cases, primary wound closure could be performed (*n* = 18, 26.5%).

#### 2.2.3. Inpatient Stay

Subjects included in this study were hospitalized for an average of 20 days. The duration of inpatient stay after a fibula transplant varied between seven and 52 days with a median of 17 days (Table 1).

### 2.3. Follow-Up Examination

#### 2.3.1. Complications at the Donor-Site

**Figure 1:**
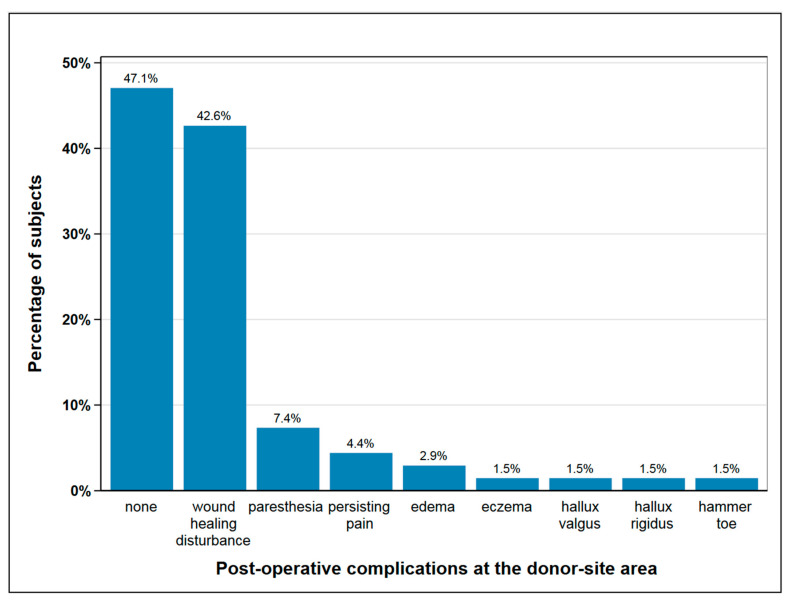
Post-operative complications at the donor-site area; seven subjects suffered from a second complication besides wound healing disturbance, which is paresthesia and persisting pain (each *n* = 2), edema, hallux rigidus and eczema (each *n* = 1); with seven cases of double complications this figure depicts *n* = 75 complications in total.

Post-operatively, 36 subjects (53%) experienced complications at the donor-site, mainly delayed wound healing and paresthesia, as shown in Figure 1 Seven subjects with wound healing disturbance suffered from a second complication, two in each case from paresthesia and persisting pain and one in each case from edema, hallux rigidus, and eczema. In 18% of subjects (*n* = 12), one or more surgical procedures were necessary at the donor site following the primary operation, mainly due to delayed wound healing and infection (Figure 1).

#### 2.3.2. Post-Operative Pain

**Figure 2:**
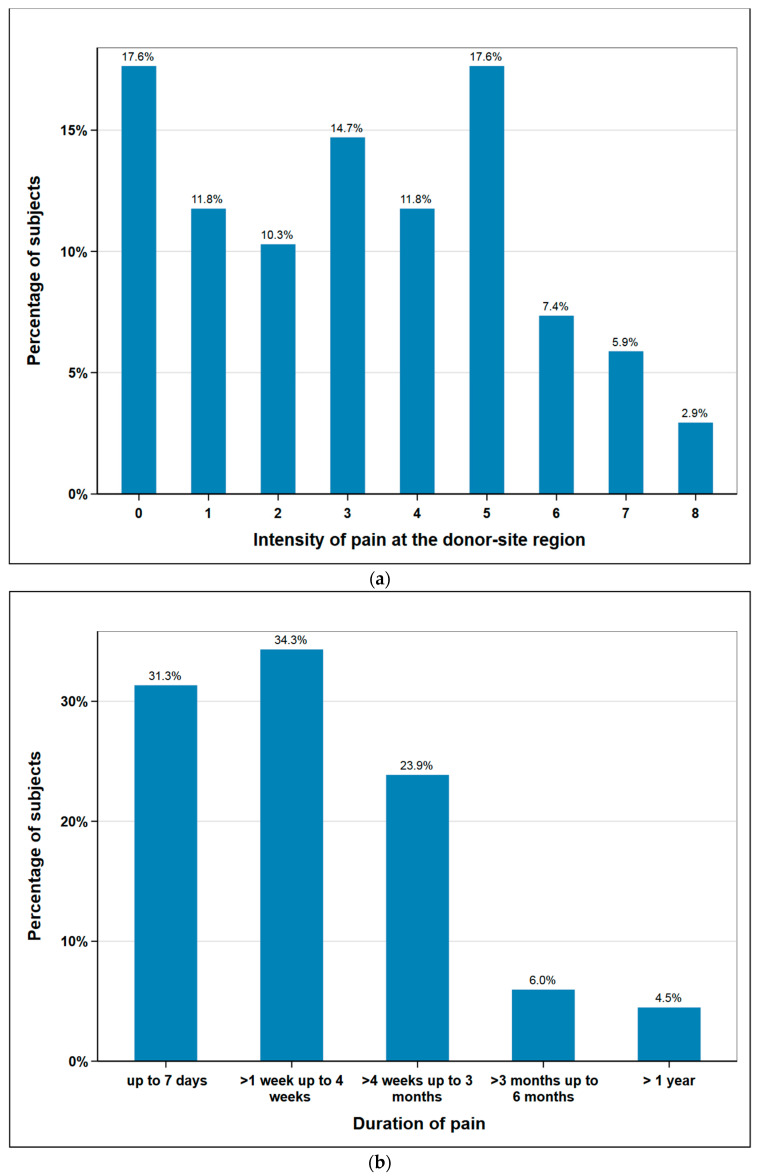
Responses of subjects regarding donor site pain: (**a**) intensity in scale 0 (no pain) to 10 (maximum imaginable pain), *n* = 68; (**b**) duration of pain (weeks, months, >1 year), *n* = 67.

The subjects rated postoperative pain sensation at the donor-site region on a numeric rating scale (NRS) from 0 to 10 (0 no pain, 10 most intense pain imaginable). The mean was 3.2 (median 3) and the maximum values 9 and 10 were not given by any patient. Additionally, post-operative pain duration was documented. 88% of the subjects were free of pain after three months at the latest. One subject stated to have persisting pain at a short follow-up time of just three months. As this subject had unfortunately died when we tried to assess the pain duration at a later date, it was excluded from this figure. Therefore, Figure 2b shows the data of *n* = 67 subjects (Figure 2).

#### 2.3.3. Fibular Nerve Function and Sensitivity Disorder

**Figure 3:**
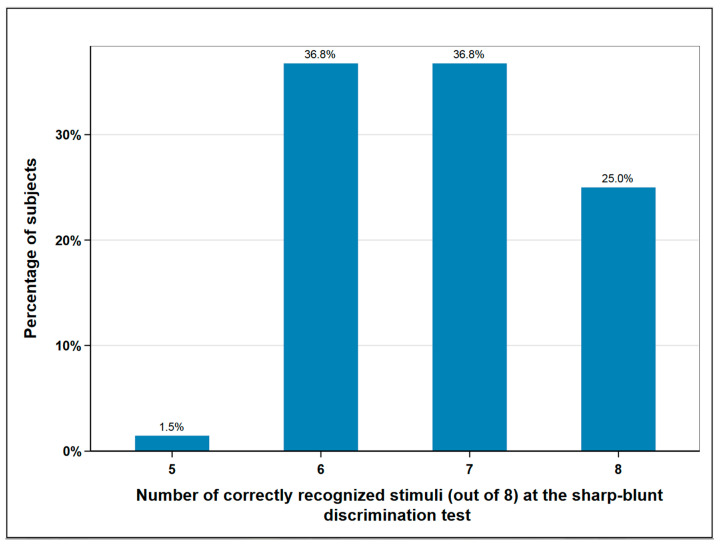
Sharp-blunt discrimination test at the donor-site; eight and seven correctly recognized stimuli correspond to normal sensation, six and lower to impaired sensation; *n* = 68.

A total of 10.3% (*n* = 7) of the subjects experienced a disturbance in the foot elevation, which indicates a lesion of the fibular nerve. The superficial sensitivity was tested in the area of the surgical scar by means of sharp-blunt discrimination test. A total of eight sharp or blunt stimuli were placed on the skin in the same area using a dental probe. Normal sensitivity was assumed if the patient was able to correctly assign seven or eight of the stimuli. Less recognized stimuli corresponded to hypesthesia. According to this classification, normal sensitivity was found in 62% (*n* = 42) of subjects and impaired sensitivity in 38% (*n* = 26) (Figure 3).

#### 2.3.4. Scar

**Figure 4:**
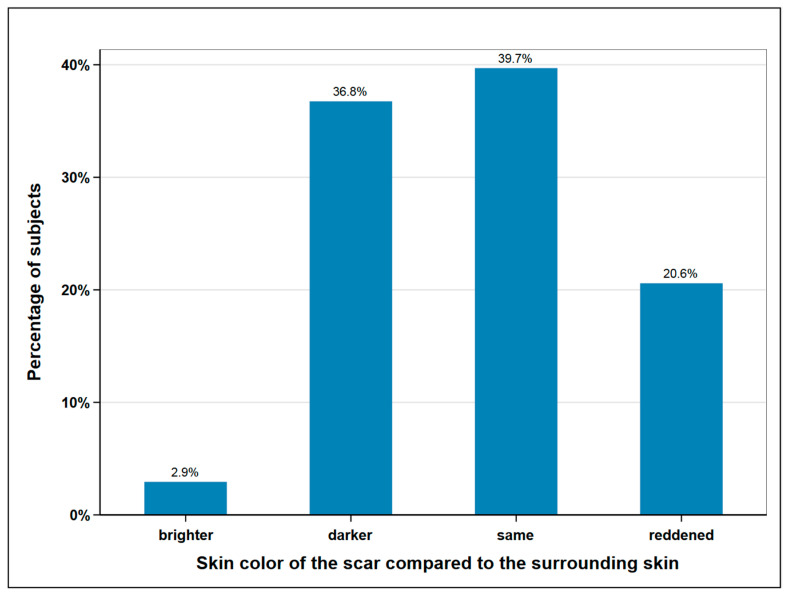
Skin color of the scar compared to surrounding skin; *n* = 68.

The size of the scar was measured with a tape measure and the skin color of the scar area was determined and documented by a picture. The mean average scar length was measured at 32.7 cm, with 40 cm being the maximum and 25 cm the minimum length. In terms of width, we distinguished between donor site scars with and without skin island removal. In the 16 subjects without skin island removal, the average width was measured at 0.63 cm. In the 52 remaining subjects, the scar area of the skin island measured at an average width of 6.4 cm. The skin color of the scar was compared to the surrounding skin and classified as lighter, darker, the same, or reddened (Figure 4).

#### 2.3.5. Level of Patient Satisfaction

**Figure 5:**
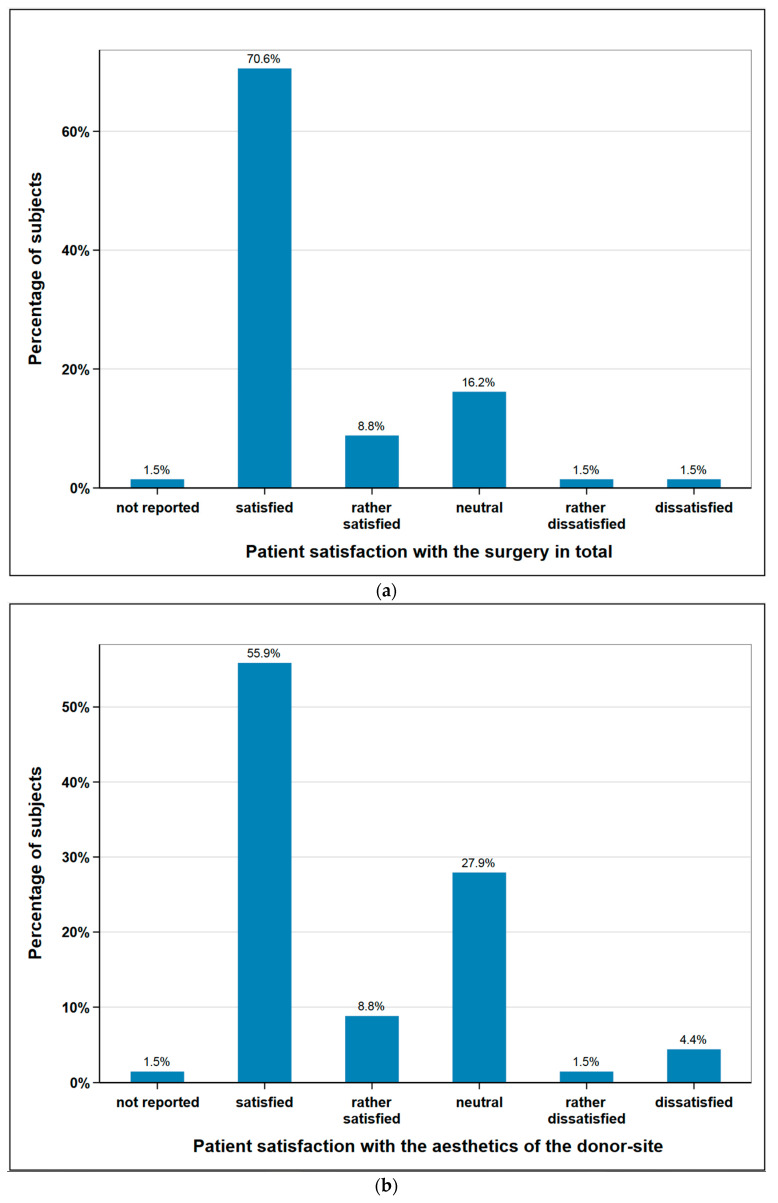
Subjects satisfaction with (**a**) the surgery in total (*n* = 68); (**b**) aesthetics of the donor-site (*n* = 68).

The subjects were asked about their satisfaction concerning the aesthetics of the donor-site and the overall operation. 64.7% of the subjects (*n* = 44) were satisfied or rather satisfied regarding aesthetics of the donor site, 5.9% (*n* = 4) were unsatisfied or rather unsatisfied, and 27.9% (*n* = 19) remained neutral. With regard the operation in general, 79.4% of the subjects (*n* = 54) were satisfied or rather satisfied, 2.9% (*n* = 2) unsatisfied or rather unsatisfied, and 16.2% (*n* = 11) remained neutral. One patient did not provide any information on either aspect (Figure 5).

### 2.4. Star Excursion Balance Test—SEBT

**Figure 6:**
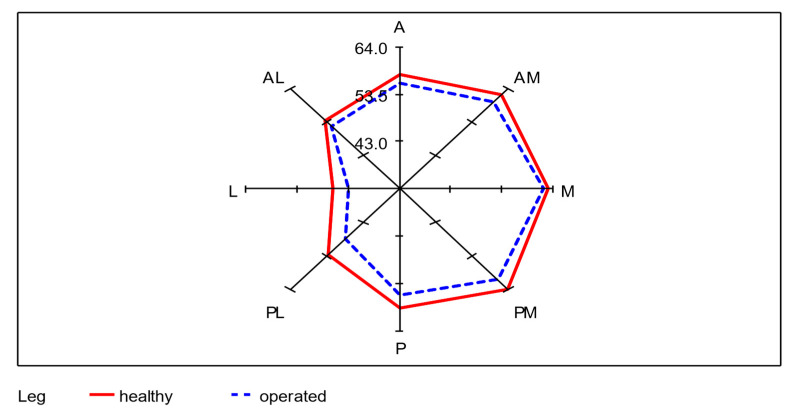
Radar plot for the visualization of the differences of the mean values between healthy and operated leg in each of the eight directions; *n* = 61.

Of the total 68 participating subjects, seven subjects were unable to perform the SEBT, even after several attempts. This was mainly due to balance problems when standing on one leg. To evaluate the SEBT data, the mean of each of the remaining 61 subjects was taken from the three values achieved per axis of movement as the starting value. The mean values of SEBT of the donor-legs and the non-operated legs from all eight directions (A, AM, M, PM, P, PL, L, AL) were calculated. An average of 55.3 cm was achieved with the operated leg as the supporting leg, which corresponds to 95.7% (0.95 confidence interval: [94.4%; 97.0%]) of 57.9 cm achieved with the healthy leg. The lowest lengths were reached in the lateral direction with both legs. A significant difference between the healthy and operated leg in all eight directions was determined using a paired *t*-test (*p* < 0.01). The differences between the average SEBT values of unoperated and operated legs for each of the 61 subjects were recorded. In the median values, the greatest lengths were measured in the medial direction for the healthy leg and in the anteromedial direction for the operated leg. As in the mean values, both legs reached the lowest lengths in the lateral direction. The mean absolute difference is largest in the posterolateral direction with 4.1 cm and smallest in the medial direction with 1.8 cm (Table 2; Figure 6).

### 2.5. Foot and Ankle Disability Index—FADI

**Figure 7:**
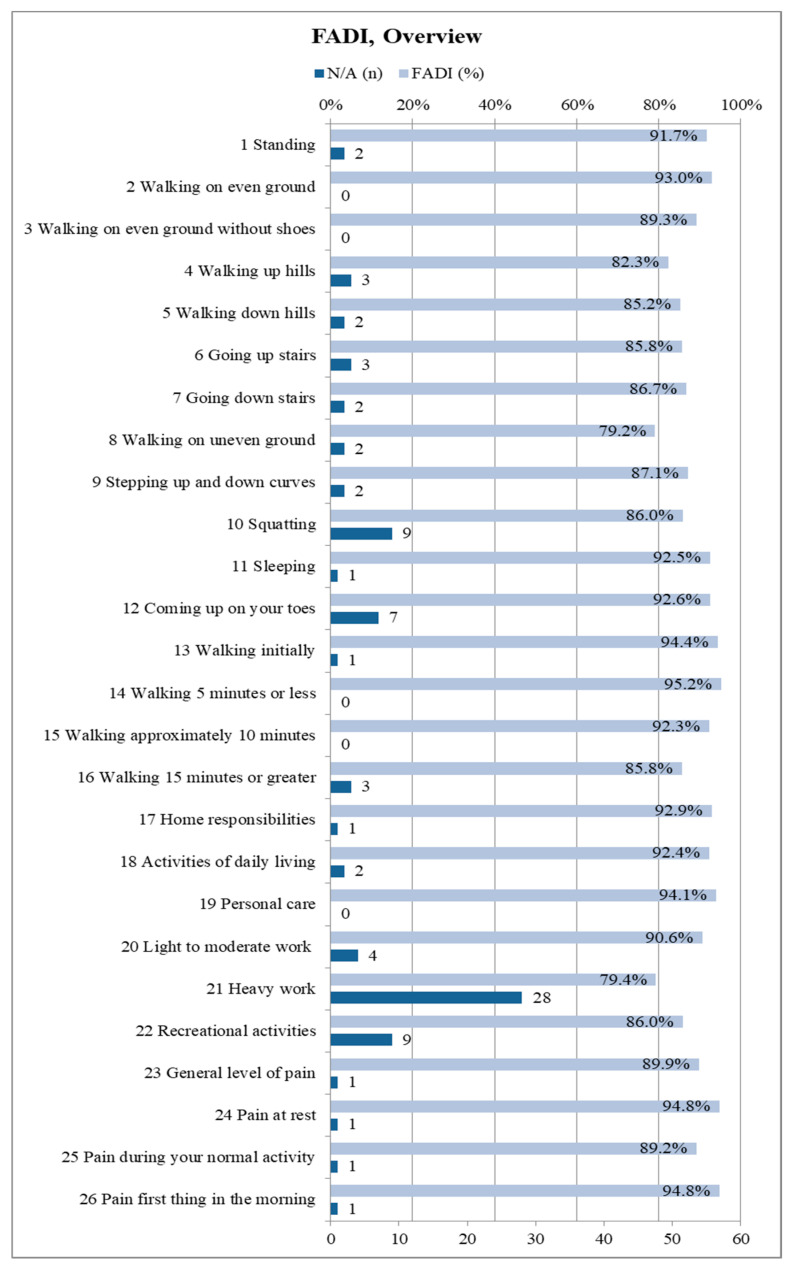
Summary of the Foot and Ankle Disability Index (FADI) results in detail for all 26 items (*n* = 68); depicted percentages are mean values; the maximum score of 100% corresponds to 4 points and represents “no difficulty/pain”, 75% corresponds to three points and represents “slight difficulty/mild pain”, 50% corresponds to two points and represents “moderate difficulty/pain”, 25% corresponds to one point and represents “extreme difficulty/severe pain”, 0% corresponds to one point and represents “unable to do/unbearable”; if subjects were limited by something else than their lower leg condition resulting from fibula flap harvest the related item is marked as “N/A” and excluded from the total score.

The FADI questionnaire included all 68 participants. A median FADI score of 96.0% (0.95-confidence interval: [92.3%; 98.1%]) was recorded (mean: 89.4%). Becasue the score only shows the overall average, but not the distribution of points for the 26 individual items, it is worthwhile to present them individually. Thus, one can see, in detail, to which extent the fibula removal affects the subjects in the respective activities as well as their pain sensation. Figure 7 illustrates an overview about each of the 26 items of the FADI questionnaire. The maximum of four points corresponds to 100% in the figure, which states “no difficulty/no pain at all”. Subjects were restricted due to other causes than fibula harvesting and were thus removed from the respective score.

27.9% of the subjects always scored the full four points on each FADI item. Accordingly, 72.1% of the subjects had some kind of limitation due to the donor site morbidity following fibula transplantation. The majority of limitations were minor (Figure 7).

## 3. Discussion

In this study, the donor-site morbidity after fibula transplantation was examined with particular focus on the extent of restrictions regarding stability and balance of the operated leg as well as quality of life of the patient. To date, the donor-site morbidity was described as low in literature. However, fluctuations between 1–19% were found in comparable studies [23].

### 3.1. Evaluation of Material and Methods

The number of 68 subjects included in this study corresponds to the values of comparable studies [31] or is significantly higher [34,38,39]. The average age of the subjects in this study was 55 years and 68% of the subjects were of male sex. Both correspond to the main indication of the fibular graft, the oral tumors and especially squamous cell carcinomas [27,31,40,41,42,43,44,45]. Examination sheets and questionnaires used in this study were obtained from similar studies that have been tried and tested in literature [23,27,46,47]. The two main parameters of the study, the SEBT and FADI, were both described as reliable tools to examine ankle injury [36,48,49,50]. SEBT was used in the sense of a “split leg” design. In each patient, the operated supporting leg served as the group to be examined, and the healthy supporting leg the control group. General differences between the left and right leg were already ruled out in literature, hence both sides can be regarded as equivalent [49,51]. In order to avoid errors due to inaccuracies in the axis cross, it was printed on a tarp. Seven subjects were unable to perform the SEBT; therefore, the number of participants was reduced to *n* = 61. A simplified variant of the SEBT, e.g., the Y-Balance Test (YBT) consisting of three direction axes, was not used due to the assumed limited significance [52]. FADI as a retrospective questionnaire has a very low error margin. All of the items of the FADI questionnaire were explained to the subjects by the examiner, any questions answered, and the questionnaires were filled out together with the examiner [35]. There are alternatives to SEBT and FADI in comparable literature. The American Orthopedic Foot and Ankle Society’s index (AOFAS) and the 36-items short form (SF-36) are the most frequently described questionnaires, but they are not validated for populations with ankle instability [26,53,54]. In addition to the FADI, the Foot and Ankle Outcome Score (FAOS), and the Foot and Ankle Ability Measure (FAAM) also meet this requirement. Because our subjects no longer practiced any sports activities, FADI seemed the most suitable method for the study subjects [54,55]. As an alternative to the SEBT, the 6-min. walk test (6MWT) has also been mentioned in literature as a physical test after fibula removal, but is only suitable to compare different collectives or a collective at different times [32,56].

In this study, pain severity was determined on a numerical scale from 0–10, which is equivalent to the visual analog scale (VAS) that was used in other studies [57,58]. However, the retrospective assessment of pain after a long follow-up time of 46 months can be inaccurate. Damage to the nerve root of the fifth lumbar nerve can be recorded using the sharp-blunt discrimination with the aid of a dental probe [59,60]. Here, further sensory checks are useful to determine the severity and location of nerve damage.

### 3.2. Discussion of the Results

The main indication for fibula transplantation in this study subjects was tumor (95,6%, *n* = 65), most of them squamous cell carcinomas with 69% (*n* = 47). In literature, the indication rates due to a tumor diagnosis are described at lower rates 62–89% [30,34,40,45,61].

#### 3.2.1. Subjects Collective and Surgical Procedures

The body mass index (BMI) in our study determined an average value of 24.3 kg/m², which is in the upper range of normal weight and it is similar to values from comparable studies [38,53,62,63]. For better comparability with other studies, we summarize our graft types to fibula grafts with skin (76%) and without skin island (24%). In similar studies, the values for grafts with a skin island are usually between 47 and 90% [27,31,64,65,66], but there is also a study with only 18% [67]. A general classification of the grafts could improve the comparability between future studies. Wound closure in this study was mainly performed using skin grafts (73.5%), and the rest was primarily sutured. In literature, the values fluctuate strongly between 11.3% [31], 31% [1], 51% [40], 64% [27], 66% [68], and 84% [39] specified for skin graft closure. The subjects in our study were hospitalized for an average of 20 days postoperatively. In the setting of primary wound closure, the average stay lasted 16 days, in the setting of a split skin graft 22 days. Only a few studies provide specific information on in-patient stays that fluctuate between 12 and 26 days [30,53,65,68,69].

#### 3.2.2. Surgical Complications

The total post-operative surgical complication rate was 53% of all study subjects. This seems to be very high when compared to 1–19% of the comparative study by Ling et al. [23], whereas individual studies also give high values of complications [34,65,70]. However, if the temporary complications, such as delayed wound healing and edema, were excluded, the long-term complication rate is 16%. In the case of temporary complications, wound healing disorders with 38% (*n* = 26) and edema with 3% (*n* = 2) are in the range of values from the literature of 4–38 and 1.5–8% [23,24,30,43]. Long-term complications reported in this study were mainly paresthesia with 7.3% (*n* = 5). In literature, higher values of 21 to 37% were described [41,71]. Unfortunately, there are no uniform categories regarding nerve injuries. Hyp-, par-, and anesthesia are often summarized as sensory deficits. A comparable study by Ling and Peng calculated an average frequency of 6.95% for sensory deficits, but the values vary between 1.7–76% [23]. Other studies reported values up to 48% [26], Shah et al. mentioned 23% (*n* = 6) for numbness in the area of the fibular and Sural nerve [34]. For hypesthesia, the sharp-blunt discrimination test in this study resulted in a value of 38%, which is higher than the results in two comparable studies (11–20%) [39,72]. In this study, damage to the fibular nerve was found in 10.3% (*n* = 7) of subjects. However, only foot lifting was examined, which makes the value susceptible to errors and it only indicated an affection of the deep fibular nerve. In literature, the values range between 1.7 to 31% [30,71,73]. In our study, persistent pain was reported at a rate of 4.4% (*n* = 3) of the subjects, which approximately corresponds to the value of 5% in Schardt et al. [39]. In literature, different data on donor-site pain can be found, varying between 0–100%. Ling and Peng et al. reported a mean pain value of 6.5%, the main source being ankle pain [23]. Further studies presented rates of 7, 9, and 21% for moderate pain [72,74,75], 43% for severe pain [72], 51% for load-dependent pain [26], and up to 73% for persistent pain [29]. A total of *n* = 3 subjects (4.4%) developed a complication affecting the toes, including hallux valgus, hallux rigidus, and hammer toe (*n* = 1 each). Hallux valgus is only reported in a comparable study, the incidence here is 16%, which seems very high [76]. Hallux rigidus is described in two studies, with values between 10 and 18% [77,78]. Hammer toes were explicitly stated in only one study (6%) [79]. Surgical revision due to persistent wound infection was necessary in 12 out of our subjects (18%), including *n* = 2 subjects (3%), who needed a second wound revision. In these subjects, skin graft was performed in 91.6% (*n* = 11). Only Sieg et al. found comparable values of 13% (*n* = 9) for a surgical revision, including *n* = 5 subjects with split skin coverage [31]. In our collective, the average pain severity was postoperatively rated on a numerical scale from 0 to 10, with an average of 3.2. In two studies, an equivalent visual analogue scale (VAS) was described at 3.7 postoperatively, and 1.3 and 1 after a minimum of one year of follow-up [39,57,62]. 88% (*n* = 60) of the subjects in this study stated lengths of up to three months at the pain duration at the donor-site area.

#### 3.2.3. SEBT und FADI

The SEBT and FADI recorded in this study show significant restrictions due to fibula flap harvesting. However, these restrictions did not seem to have any serious disadvantages for subjects. The SEBT had an average difference of 2.6 cm or 4.5% between the reach of a healthy and operated leg. A comparison of the differences in length of the SEBT to values from literature can be found in the following overview that is delineated in Table 3.

The average differences between affected and healthy leg in SEBT in this study were comparable to other publications (2.2 to 8.8%) [84,86]. However, only five other studies had a split-leg design and the SEBT values were completely measured for all eight directions [34,36,81,82,84]. Interestingly, the only study that did not report significant differences between the involved and uninvolved limbs was the study of Shah et al., which also examined donor-site morbidity after fibula transplantation [34]. Only in the study published by Kobayashi et al., the distribution of values is similar to ours with the greatest differences in the posterior (P), posterolateral (PL), and lateral (L) directions [86]. The study by Karagiannakis et al., in which electromyography during SEBT was used, showed that in the three aforementioned directions, there is significantly higher activity. The fibularis brevis muscle has a connection to the fibular bone; in addition, both muscles could also be restricted by lesions of the deep fibularis and superficialis nerve [88]. This could explain the large differences in the posterior, posterolateral, and lateral directions [32,89]. With the exception of the study by Shah et al., the main cause of the different SEBT results is likely to be the younger collectives and different medical conditions. Chronic ankle instability, in particular, seems to have a similar impact on SEBT as a fibula transplant.

In our study the subjects achieved 89.4% of the FADI, which corresponds to values for the chronic ankle instability (CAI—Chronic Ankle Instability). This indicates an average of 89.3% for the FADI. Shah et al. also give similar values for the FADI, with 89% after fibula transplantation [34]. The following overview Table 4 shows a comparison with selected literature:

In general, it turns out that our FADI averages correspond to studies on chronic ankle instability (CAI). However, our collective is significantly older than in the CAI studies, which mainly examine young and athletic subjects. Only the study conducted by Shah et al. resembles our study with regard to the average age of 46.4 years and the FADI of 89%, but the SEBT values stand in stark contrast to our values.

### 3.3. Limitations of This Study

The retrospective design and the relatively high drop-out rate are limitations of the study. As the patient questionnaires were filled out years after the surgical procedure, the accuracy and, hence, validity can be limited.

## 4. Materials and Methods

### 4.1. Study Design and Examiner Blinding and Calibration

This study was conducted as a retrospective clinical split-leg study at the University Hospital Giessen, Germany, Department for Oral and Maxillofacial Surgery. One examiner was responsible for the entire investigation and data acquisition. Previous training and calibration for the examiner was performed prior to patient examination.

### 4.2. Patient Inclusion and Exclusion Criteria

All of the subjects who underwent fibula transplantation for orofacial reconstruction between January 2002 and November 2017 were included in the study. The subjects who needed walking aids were excluded as the only exclusion criteria. Because these were mainly cancer subjects, an increased drop-out rate due to deceased and physically impaired subjects was to be expected. A number of 68 subjects could be recruited for the study.

### 4.3. Study Parameters

The basic and surgical data, such as patient age, sex, and diagnosis, were taken from existing files; in individual cases, the data was verified with the patient during the follow-up examination. The study parameters were assessed in one follow-up examination per subject. These included the permanent complications such as pain and nerve lesions. BMI classified according to the world health organization (WHO) classification to: underweight below 18.5, normal weight 18.5–24.9, pre-obesity 25.0–29.9, obesity grade I 30.0–34.9, and obesity grade II 35.0–39.9. Table 5 summarizes all documented data.

### 4.4. Star Excursion Balance Test—SEBT

The Star Excursion Balance Test (SEBT) is mainly used in orthopedics and sports medicine to determine ankle function/leg function [97]. It was first described in its current form in 2000 by Hertel et al. [98]. Previously, it had already been described in a simplified form as a “star excursion test” with four instead of eight movement axes [99]. It can be used to record various restrictions, diseases, and injuries to the lower extremity and ankle [49]. It is often used in the assessment of ankle injuries, particularly chronic ankle instability (CAI—Chronic ankle instability), where it has a high degree of reliability [35,37,49,100]. This is of particular importance for the present study, since the fibula is directly involved in the upper ankle joint. Overall, the Star Excursion Balance Test is widely described in literature as a reliable method for determining the stability of the lower extremity and the ankle [48,51,99]. The SEBT measures the maximum range of one leg while standing on the other one. The subject stands in the middle of an eight-section cross, with all of the axes orientated at an 45° angle to each other. The axes are referred to clockwise from the right leg, counterclockwise from the left leg, as anterior (A), anterolateral (AL), lateral (L), posterolateral (PL), posterior (P), posteromedial (PM), medial (M), and anteromedial (AM). During the test, the complete balance must be kept on the supporting leg, the other leg must not carry any weight. The subject tries to move the contralateral leg as far as possible in each of the eight directions without losing balance. The examiner marks the furthest point where the foot touches the axis. These values are marked for each of the eight axes, with a total of three passes per left and right leg. A slightly adapted variant of the SEBT was used in the study [101]. The test setup was printed on a tarp for better feasibility.

**Figure 8:**
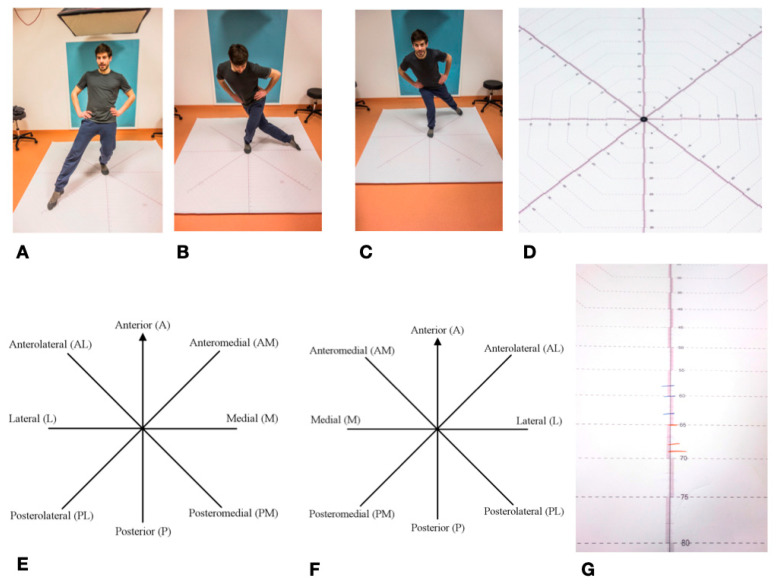
Star Excursion Balance Test—SEBT: (**A**–**C**) Mainstay left, towards Posterolateral; Lateral; and, Posteromedial; (**D**) SEBT detailed view of measuring cross; (**E**) SEBT stand on the left; (**F**) SEBT mainstay on the right; and, (**G**) Length marking as an example; blue = right leg; red = left leg.

All eight directions were continuously scaled to a length of 100 cm and the individual values were easy to read. After the subjects had been informed and a demonstration had taken place, the foot of the supporting leg was placed centrally on the center of the cross in the anterior direction. This was followed by four practice runs for each leg. Thereafter, starting with a randomly selected standing leg, three runs were carried out per leg with the standing leg being changed after each round. The achieved lengths were noted on the axes with a wipeable marker in blue (right leg) and red (left leg). The values, rounded to the nearest centimeter, were then noted in a table. The mean value was calculated from the three values noted for each axis and supporting leg, which resulted in 16 values for eight axes and two legs. These values were used for the subsequent statistical analysis SEBT set as the primary endpoint in this study (Figure 8).

### 4.5. Foot and Ankle Disability Index—FADI

The Foot and Ankle Disability Index (FADI) was first mentioned in literature in 1999 [55]. It is a questionnaire, in which subjects with ankle injuries can assess their injury-related limitation in terms of movement, everyday activities, and pain. Similar to the SEBT, the FADI is particularly suitable for the assessment of chronic ankle instability (CAI) [102]. Therefore, it is often used in studies with the SEBT and it is particularly suitable for evaluating rehabilitation after ankle injuries [35,90,103]. In addition to the basic form of the FADI, there is an extension consisting of eight additional questions regarding sport activities, the so-called “FADI Sport”.

The basic form of the Foot and Ankle Disability Index (FADI) consists of 26 items. For 22 of these questions, the patient has to rate different activities of daily life (ADL) and the following points can be assigned: four points (no difficulty), three points (slight difficulty), two points (moderate difficulty), one point (extreme difficulty), or 0 points (unable to do). The four remaining questions are about pain intensity; here the points can be assigned, as follows: four points (no pain), three points (slight pain), two points (moderate pain), one point (severe pain), 0 points (unbearable). Overall, a maximum score of 104 points (26 × 4 points) is possible. It is particularly important that the only cause of the complaints must come from the disease or injury to the ankle. If another illness is the cause of the restrictions or pain, the respective question is marked with “N/A” and taken off the total score and the total score is adjusted. In this case, the examined disease or injury to the ankle corresponded to the raising of the fibular graft. The original test can be expanded to include the “FADI Sport” subscale for a young and active athletic collective, in which eight questions about sporting activities have to be answered in the same scheme [50]. The structure of the questionnaire used was taken from Hale et al. [35]. The FADI questionnaire was explained to each patient by the examiner, who took special care that each patient understood the questions and above-mentioned assessment requirements. The questions were answered together with the patient, and the score was noted. The overall result was then determined in absolute terms and as a percentage value.

### 4.6. Ethics, Privacy and Statistical Analysis

The ethics committee at the Medical Faculty of Justus-Liebig University Giessen approved the study under the approval number 232/14. The collected patient data were pseudonymized before statistical analysis was performed. Each patient could only be assigned to the respective data record using a key. The statistical evaluation was carried out together with the Institute for Medical Informatics at University Hospital Giessen, Germany. Continuous variables are expressed as mean ± standard deviation (std), median, minimum, and maximum, categorical variables are reported as absolute and/or relative frequencies. Normal distribution of continuous variables were evaluated by QQ-Plot, group comparison was executed by *t*-test. No adjustment for multiple testing was performed. All obtained parameters were listed in a combined Excel table and then evaluated using SAS (version 9.4 SAS Institute, Cary, NC, USA).

## 5. Conclusions

Donor-site morbidity after fibula transplantation is relatively low and, compared to the outstanding benefits of the fibula graft in the reconstructive facial surgery, the complications are still well justifiable. The vast majority of subjects are satisfied with both the functional and aesthetic result. The most common complications identified were transient wound healing disorders as well as paresthesia.

The examinations using SEBT and FADI showed a small but significant, lower stability, and ankle function due to the operation, similar to a chronic ankle instability (CAI). However, the limitations of quality of life and daily activities were minimal for most subjects. SEBT and FADI both seem to be suitable methods to examine patients after fibula transplantation. In general, future studies on donor-site morbidity after fibula transplantation should use uniform test methods. This is the only way to effectively compare the abundant data in literature.

## Figures and Tables

**Table 1:** A demographics table illustrate the collected data and subject population. Data are given as mean (standard deviation) or as percentage % (absolute frequency).

Variable	Mean (Standard Deviation) or Percentage % (Absolute Frequency)
Age (years) at surgery	55.4 (12.5)
Gender (m/f)	67.6% (*n* = 46)/32.4% (*n* = 22)
Height (cm) at surgery	174 (11)
Weight (kg) at surgery	74 (17)
BMI at surgery	
underweight	8.8% (*n* = 6)
normal weight	54.4% (*n* = 37)
pre-obesity	25.0% (*n* = 17)
obesity grade I	8.8% (*n* = 6)
obesity grade II	2.9% (*n* = 2)
Indication of fibula transplantation	
squamous cell carcinoma	69.1% (*n* = 47)
ameloblastoma	8.8% (*n* = 6)
keratocystic odontogenic tumor	7.4% (*n* = 5)
jaw atrophy	2.9% (*n* = 2)
adenoid cystic carcinoma	2.9% (*n* = 2)
acinar cell carcinoma	1.5% (*n* = 1)
adenosquamous carcinoma	1.5% (*n* = 1)
mucoepidermoid carcinoma	1.5% (*n* = 1)
myoepithelial carcinoma	1.5% (*n* = 1)
hemangiopericytoma	1.5% (*n* = 1)
osteoradione necrosis	1.5% (*n* = 1)
Operated Leg (left/right)	25% (*n* = 17)/ 75% (*n* = 51)
Type of transplant	
osseomyocutan	76.5% (*n* = 52)
osseomuscular	14.7% (*n* = 10)
prefabricated	8.8% (*n* = 6)
Wound closure	
graft from thigh region	73.5% (*n* = 50)
primary sutured	26.5% (*n* = 18)
Hospitalisation time	
up to 1 week	1.5% (*n* = 1)
>1 up to 2 weeks	35.3% (*n* = 24)
>2 up to 3 weeks	27.9% (*n* = 19)
>3 up to 4 weeks	14.7% (*n* = 10)
>4 up to 5 weeks	13.2% (*n* = 9)
more than 5 weeks	7.4% (*n* = 5)

**Table 2:** Distribution of the Star Excursion Balance Test (SEBT) values (mean of three measurements) (cm), operated versus healthy legs (*n* = 61).

Direction	Operated Leg				Healthy Leg				Difference Operated—Healthy Leg				*p* Value ^1^
	Min	Max	Mean	Std	Min	Max	Mean	Std	Min	Max	Mean	Std	
A	39.3	78.0	56.7	8.1	41.0	81.0	58.8	8.5	−15.0	9.7	−2.1	3.9	0.001
AM	40.3	81.0	60.8	8.7	46.0	82.7	62.8	9.0	−18.0	8.2	−2.0	4.6	0.001
M	39.7	82.7	62.1	8.5	43.7	85.0	63.9	8.6	−15.0	8.7	−1.8	4.0	<0.001
PM	44.0	87.0	61.8	9.4	41.2	89.3	64.6	9.4	−14.0	7.7	−2.7	4.3	<0.0001
P	22.3	89.3	56.3	11.1	27.5	90.3	59.5	11.1	−11.7	7.3	−3.2	4.0	<0.0001
PL	23.7	75.7	48.9	11.5	21.3	86.0	53.0	12.1	−22.0	7.7	−4.1	5.9	<0.0001
L	21.7	70.7	43.6	8.3	20.7	69.0	46.5	8.5	−19.0	8.0	−2.8	4.4	<0.0001
AL	36.7	77.0	52.3	7.9	37.0	78.3	54.2	8.0	−14.0	11.0	−2.0	4.5	0.001

^1^ paired *t*-test.

**Table 3:** SEBT literature comparison, percentage differences between affected leg and control group; SEBT = overall percentage difference, mean value; A, AM, M PM, P, PL, L, AL = differences percentages for each reach direction, mean value; CAI = chronic ankle instability; AD = ankle distortion; CL = cruciate ligament surgery (Lig. cruciforme anterior); FT = fibula transplantation SL = “Split Leg” (comparison between legs of the same person).

Literature Review	Age (y)	SEBT Ø (%)	A (%)	AM (%)	M (%)	PM (%)	P (%)	PL (%)	L (%)	AL (%)
Olmsted 2002 *n* = 20 CAI, SL [36]	19.8	5.5	3.9	5.4	3.8	6.3	3.8	3.1	10.6	4.2
Gribble 2004 *n* = 30 CAI [80]	22.3	-	5.6	-	5.9	-	7.4	-	-	-
Hertel 2006 *n* = 48 CAI, SL [81]	20.9	3.3	3.8	2.4	3.4	4.5	2.4	2.5	4.3	2.8
Hale 2007 * n = 29 CAI,SL [82]	21.4	4.2	2.8	3.9	3.5	4.7	3.2	5.2	6.4	3.8
Delahunt 2013 *n* = 17 CL [83]	20.8	-	3.8	-	-	8.6	-	9.4	-	-
Kalichmann 2016 *n* = 20 *AD,* SL [84]	25.4	2.2	2	2.4	1.6	3.7	2.8	1.7	0.6	3
Ko 2018 *n* = 24 CAI, SL [85]	15.5	-	11.3	-	-	9.4	-	7	-	-
Kobayashi 2019 *n* = 50 *CAI* [86]	20.8	8.8	7.3	7.1	7.2	7.7	9.7	13	10	8.3
Hadadi 2019 *n* = 44 CAI [87]	22.9	-	-	5.3	9.3	6.4	-	-	-	-
average	*22*	*5.1*	*5.8*	*4.5*	*5.2*	*6.3*	*5.2*	*6.3*	*6.4*	*4.6*
our study *n* = 61	55.4	4.5	3.6	3.2	2.8	4.2	5.4	7.8	6.1	3.6
Shah 2017 * n = 26 FT SL [34]	49.4	−0.2	−1.6	3.1	−1.6	−3.3	0	0	1.6	0

* = FADI also used.

**Table 4:** FADI literature comparison; FADI = Foot and Ankle Disability Index; CAI = Chronic ankle instability; score shows mean value of all 26 FADI items as percentage; CAI average = mean value of all depicted studies involving subject with CAI; FADI average = overall mean value of all compared studies.

Literature Review	Medical Condition	*n*	Age (y)	Score (%)
Hale & Hertel 2005 [35]	CAI	30	21.5	89.6
Hale 2007 * [82]	CAI	29	21.4	89.7
McKeon 2008 [90]	CAI	31	20.9	84.2
Cook 2010 [91]	severe arthritis	79	63.9	46
Hubbard-Turner 2012 [92]	CAI	120	20.6	87.6
Wikstrom 2012 [93]	CAI	24	21.7	95.2
Kim 2013 [94]	professional athletes	85	19.8	88.9
Shah 2017 * [34]	Fibula Transplant	26	46.4	89
Sanders 2019 [95]	Fibula fracture surgery	103	39.5	91.4
Carter 2019 [96]	Tibia Pilon fracture	99	-	76
CAI average	CAI	46,8	21.2	89.26
FADI average		62,6	30.6	83.8
Our study *	Fibula transplant	68	55.4	89.4

* = SEBT also used.

**Table 5:** The collected study parameters divided into basic, surgical, and follow-up data.

Basic Data	Surgical Data	Follow-Up Examination
	Indication for surgery	Subjective pain intensity (harvested leg)
Follow-up time		Duration of pain
Age of the patient (surgery)	Localisation of the harvested flap (right or left leg)	Fibular nerve lesion
	Type of graft removed (osseocutaneus, osseomuscular, prefabricated)	blunt sharp discrimination test (harvested leg)
Height	Type of wound closure	Scar length and width (harvested leg)
Body weight	Hospitalization time	Skin color (harvested leg)
Body Mass Index (BMI)	Post-operative complications	Subjects satisfaction aesthetics (harvested leg)
		Subjects general satisfaction
